# NF-*κ*B-Related Metabolic Gene Signature Predicts the Prognosis and Immunotherapy Response in Gastric Cancer

**DOI:** 10.1155/2022/5092505

**Published:** 2022-01-04

**Authors:** Qiuxiang Chen, Xiaojing Du, Sunkuan Hu, Qingke Huang

**Affiliations:** ^1^Department of Ultrasonic Imaging, The First Affiliated Hospital of Wenzhou Medical University, Wenzhou, Zhejiang 325035, China; ^2^Department of Gastroenterology, Minhang Hospital, Fudan University, 170 Xinsong Road, Shanghai 201199, China; ^3^Department of Gastroenterology, The First Affiliated Hospital of Wenzhou Medical University, Wenzhou, Zhejiang 325035, China

## Abstract

**Background:**

Sufficient evidence indicated the crucial role of NF-*κ*B family played in gastric cancer (GC). The novel discovery that NF-*κ*B could regulate cancer metabolism and immune evasion greatly increased its attraction in cancer research. However, the correlation among NF-*κ*B, metabolism, and cancer immunity in GC still requires further improvement.

**Methods:**

TCGA, hTFtarget, and MSigDB databases were employed to identify NF-*κ*B-related metabolic genes (NFMGs). Based on NFMGs, we used consensus clustering to divide GC patients into two subtypes. GSVA was employed to analyze the enriched pathway. ESTIMATE, CIBERSORT, ssGSEA, and MCPcounter algorithms were applied to evaluate immune infiltration in GC. The tumor immune dysfunction and exclusion (TIDE) algorithm was used to predict patients' response to immunotherapy. We also established a NFMG-related risk score by using the LASSO regression model and assessed its efficacy in TCGA and GSE62254 datasets.

**Results:**

We used 27 NFMGs to conduct an unsupervised clustering on GC samples and classified them into two clusters. Cluster 1 was characterized by high active metabolism, tumor mutant burden, and microsatellite instability, while cluster 2 was featured with high immune infiltration. Compared to cluster 2, cluster 1 had a better prognosis and higher response to immunotherapy. In addition, we constructed a 12-NFMG (*ADCY3*, *AHCY*, *CHDH*, *GUCY1A2*, *ITPA*, *MTHFD2*, *NRP1*, *POLA1*, *POLR1A*, *POLR3A*, *POLR3K*, and *SRM*) risk score. Followed analysis indicated that this risk score acted as an effectively prognostic factor in GC.

**Conclusion:**

Our data suggested that GC subtypes classified by NFMGs may effectively guide prognosis and immunotherapy. Further study of these NFMGs will deepen our understanding of NF-*κ*B-mediated cancer metabolism and immunity.

## 1. Introduction

Gastric cancer (GC) ranks as the fifth most common cancer and the fourth leading cause of cancer-related deaths worldwide, as well as an important barrier to increasing life expectancy [1]. Though the popularity using of endoscopy improved the early detection of GC, a great number of patients were still diagnosed at late stage, associated with poor outcome [2]. Hitherto, platinum or/and fluorouracil-based chemotherapy remains the first-line treatment for GC, while patients with late-stage GC still suffered poor outcome with a median overall survival (OS) being nearly 1 year [3]. The rapid boost of immunotherapy renovated the oncotherapy in the recent decade. Several immune checkpoint inhibitors (ICIs), such as Nivolumab and Pembrolizumab, have been approved by the FDA for GC treatment. Unfortunately, however, only a small cohort of patient could benefit from these treatments [4, 5]. Intratumoural heterogeneity may be responsible for the different individual response to the treatment [6]. Traditional typing is difficult to penetrate into GC's heterogeneity, and whereby further molecular research is necessary and urgent for treatment and prognostic determination.

The NF-*κ*B family, comprised of five members: NF-*κ*B1 (p50), NF-*κ*B2 (p52), c-Rel, RelA (p65), and RelB, is a well-known transcription factor family which regulates a large number of target genes and plays crucial roles in cancers including GC [6–8]. NF-*κ*B signaling pathway directly and indirectly controls key cancer hallmarks, such as cell proliferation and survival, epithelial-mesenchymal transformation (EMT), angiogenesis [8]. Novel identified hallmarks, immune evasion, and abnormal metabolism attracted the great attention of oncologists [9]. Complex tumor microenvironment conferred the complicated entanglement between cancer immunity and metabolism [10]. Recent study indicated that the canonical NF-*κ*B subunits c-Rel and RelA played a key role in the identity and function of regulatory T cells (Tregs, a type of T cell with inhibition activity against antitumor immune responses) [11]. The RelA-mediated pathway also inhibited the ubiquitination and degradation of PD-L1 [12]. In addition, several study showed that TP53 status in cancer determined RelA's affection on oxidative phosphorylation (OXPHOS). On the one hand, RelA upregulated the expression of synthesis of cytochrome c oxidase 2 (SCO2) and thereby sustained OXPHOS in wild-type TP53 expressing cancer cells [13]. On the other hand, RelA accompanied with mortalin could translocate to the mitochondria and whereby repressed OXPHOS in mutant TP53 expressing cancer cells [14]. These study emphasized the potential role of NF-*κ*B transcription factors in cancer immune evasion and abnormal metabolism.

However, whether NF-*κ*B transcription factors mediated cancer metabolism can affect GC immune microenvironment needs further exploration. A nuanced evaluation of NF-*κ*B-related metabolism and cancer immunity may reveal novel cancer vulnerabilities upon which may improve GC's immunotherapy response. Here, we hypothesized that NF-*κ*B-mediated abnormal metabolism may be involved in the regulation of GC's immune microenvironment. To this end, NF-*κ*B-related metabolic genes (NFMGs) were identified by combined analysis of The Cancer Genome Atlas (TCGA), the Molecular Signatures Database (MSigDB), and hTFtarget database. And then, we employed multiple algorithms to investigate the prognostic value of NFMGs and their correlation with GC's immune microenvironment.

## 2. Materials and Methods

### 2.1. Data Source and Differentially Expressed NFMGs

GC RNA-sequencing (RNA-seq) data (32 normal and 375 tumor) were downloaded from TCGA database (https://gdc-portal.nci.nih.gov/), among which 51 tumor RNA-seq data were excluded for incomplete clinical information. In addition, 300 GC samples (GSE62254) [15] with survival data were from the Gene Expression Omnibus (GEO) database (https://www.ncbi.nlm.nih.gov/geo/). The clinical characteristics of GC samples in TCGA and GSE62254 were displayed (Table 1).

To identify differentially expressed NFMGs, “limma” package of R (version 4.1.0) was used to screen the differentially expressed genes (DEGs) between normal and GC tissues [16]. Pearson correlation analysis was conducted between NF-*κ*B transcription factors (*NFKB1*, *NFKB2*, *REL*, *RELA*, and *RELB*) and DEGs. NF-*κ*B-related DEGs were selected according to the cutoff of ∣*R* | >0.3 and *p* < 0.001. NF-*κ*B-targeted genes were obtained from the hTFtarget database (http://bioinfo.life.hust.edu.cn/hTFtarget#!/) [17]. Furthermore, Kyoto Encyclopedia of Genes and Genomes (KEGG) gene set was downloaded from the MSigDB database, and metabolism-related pathways were selected [18, 19]. Metabolism genes (*n* = 948) were distinguished from these pathways. The overlapped genes among these parts were considered as the NFMGs.

### 2.2. NF-*κ*B Expression and Prognostic Analysis

The TIMER 2.0 dataset was employed to estimate the expression of NF-*κ*B transcription factors (*NFKB1*, *NFKB2*, *REL*, *RELA*, and *RELB*) in various cancer types [20]. The Human Protein Atlas (HPA) database (https://www.proteinatlas.org/) was used to evaluate the protein expressions of NF-*κ*B transcription factors in normal and tumor tissues. To investigate the prognostic value of NF-*κ*B transcription factors in GC, related survival analysis was conducted by using the Kaplan-Meier Plotter online tool [21].

### 2.3. NFMG-Based Consensus Clustering

At first, the least absolute shrinkage and selection operator (LASSO) algorithm and recursive feature elimination (RFE) algorithm were adopted to shrink the number of NFMGs, achieved by “glmnet” and “caret” package of R, respectively [22, 23]. The shared genes from two algorithms were served as candidate NFMGs. Consensus clustering was realized by using “ConsensusClusterPlus” package (http://www.bioconductor.org/) of R based on the candidate NFMGs. The expression of NFMGs between two clusters was visualized by using “pheatmap” package (https://cran.r-project.org/package=pheatmap). The survival analysis was performed by “survival” package (https://cran.r-project.org/package=survival).

### 2.4. KEGG and Gene Ontology (GO) Analyses

KEGG and GO analyses of NFMGs were performed by using “clusterProfiler” package of R [24] and visualized by applying “ggplot2” package (http://cran.r-project.org/package=ggplot2).

### 2.5. Gene Set Variation Analysis (GSVA)

GSVA was employed to quantify the involvement of KEGG pathways of each sample using “GSVA” package of R software [25].

### 2.6. Immune Microenvironment Estimation

The immune microenvironment estimation was achieved by multiple algorithms. The stromal score, immune score, ESTIMATE score, and tumor purity of each sample were calculated by “estimate” R package [26]. The abundance of infiltrated immune cells in each sample was estimated by CIBERSORT, Single-sample Gene Set Enrichment Analysis (ssGSEA), and MCPcounter algorithms [18, 27, 28]. Furthermore, the response to ICI was assessed by applying the tumor immune dysfunction and exclusion (TIDE) algorithm [29].

### 2.7. Tumor Mutant Burden (TMB) Estimation

TMB indicates the total number of mutations in the coding region of the evaluated gene in the tumor cell genome. The TMB of each sample was calculated by employing “TCGAmutations” package of R [30].

### 2.8. Construction of NFMG Signature with Prognostic Value

To confirm NFMGs with prognostic signature, the pairwise relationships of the candidate NFMGs were evaluated by the STRING (https://string-db.org/) online tool [31]. The cutoff for confidence scores of interactions is 0.4. MCODE app of Cytoscape (version 3.8.2) was employed to select the subclusters of the coexpression networks with the default settings (node score cutoff: 0.2 and K-core: 2), aiming to analyze the physical relationships among these distance-related genes [32, 33]. The genes in subclusters were selected as the hub NFMGs. The LASSO algorithm was applied to construct risk score (RS) for predicting patients' OS.

### 2.9. Decision Curve Analysis (DCA)

DCA was an ideal tool to calculate the clinical net benefit of each model compared to all or none strategies [34]. It was employed to judge the efficacy of mentioned RS.

### 2.10. Establishment of Nomogram

Based on RS and stage, the nomogram was constructed by using the “rms” package (https://cran.r-project.org/package=rms). The efficacy of nomogram was validated by applying calibration plots.

### 2.11. Statistical Analysis

All statistical analyses were completed by using the R software (version 4.1.0). The *t*-test was used for comparing normally distributed data, and Mann–Whitney test was for nonnormally distributed data. Continuous variables are shown as the mean ± standard deviation (SD). OS analyzed by the log-rank test meant the time from diagnosis to the last follow-up or death. If not specified above, *p* < 0.05 was regarded as statistically significant. The main code used in the R software was also uploaded (Supplemental file 1).

## 3. Results

### 3.1. NF-*κ*B Transcription Factors Were Upregulated in GC

**Figure 1:**
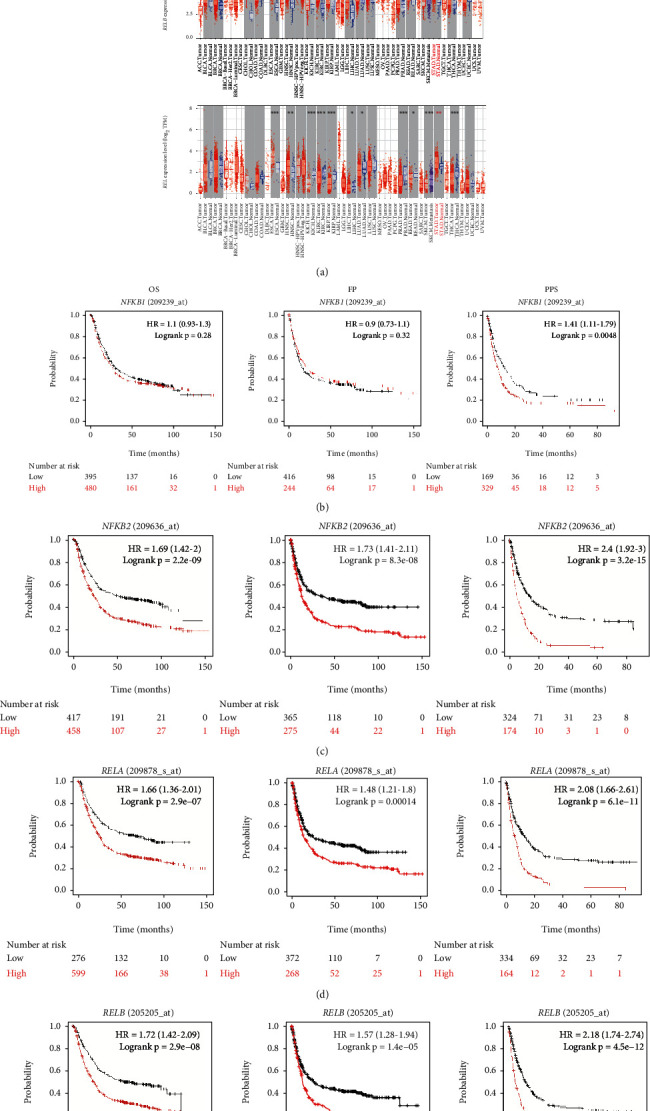
The expression of NF-*κ*B family across various cancer types. (a) The expression of *NFKB1*, *NFKB2*, *RELA*, *RELB*, and *REL* in different cancer types was determined by the TIMER 2.0 database. The abbreviations of cancer types were from TCGA database. (b–f) Survival analysis of *NFKB1*, *NFKB2*, *RELA*, *RELB*, and *REL* in GC was achieved by the Kaplan-Meier Plotter online tool. ^∗^*p* < 0.05, ^∗∗^*p* < 0.01, ^∗∗∗^*p* < 0.001, and ^∗∗∗∗^*p* < 0.0001. OS: overall survival; FP: first progression; PPS: postprogression survival; GC: gastric cancer.

At first, the expression of NF-*κ*B transcription factors in various cancer types was retrieved in the TIMER 2.0 database. As shown, the expression of *NFKB1*, *NFKB2*, *REL*, *RELA*, and *RELB* varied in different cancer types (Figure 1(a)). In some cancer types, such as cholangiocarcinoma (CHOL), esophageal carcinoma (ESCA), and head and neck squamous cell carcinoma (HNSC), all of NF-*κ*B transcription factors were upregulated, while in some cancer types, such as colon adenocarcinoma (COAD), skin cutaneous melanoma (SKCM), and uterine corpus endometrial carcinoma (UCEC), different NF-*κ*B transcription factors showed contradictory expression. This contradiction may be concerned in NF-*κ*B's bidirectional regulation of tumor [6, 8, 35, 36]. In GC, *NFKB1*, *NFKB2*, *REL*, *RELA*, and *RELB* were significantly upregulated in tumor tissues (Figure 1(a)). Immunohistochemistry data from the HPA database showed that positive expression of NF-*κ*B1, NF-*κ*B2, c-Rel, RelA, and RelB could be observed in GC tissues (Figure S1). GC patients with high expression of *NFKB2*, *REL*, *RELA*, and *RELB* possessed a significantly shorter OS and first progression (FP) than patients with low expression of these four genes, while similar significance has not been observed for *NFKB1* (Figures 1(b)–1(f)). All NF-*κ*B transcription factors have a significant predictive significance with regard to the postprogression survival (PPS) in GC: the higher expression of NF-*κ*B, the lower PPS (Figures 1(b)–1(f)). These data suggested the close connection of NF-*κ*B family with GC and its prognosis.

### 3.2. Identification of NFMGs

**Figure 2:**
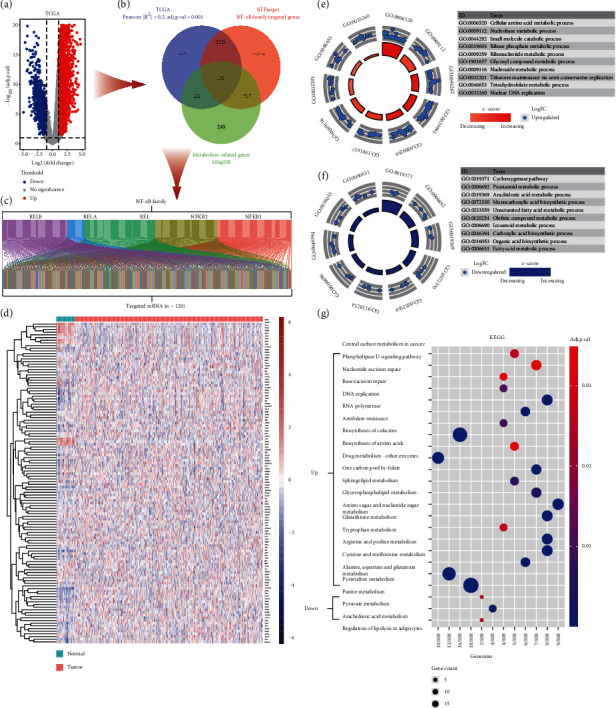
Differential expressed NFMGs in TCGA and their function analysis. (a) The volcano plot showed the DEGs in TCGA database. (b) The overlapped genes among TCGA, hTFtarget, and MSigDB databases were considered as the differential expressed NFMGs. (c) The Sankey plot displayed the targeted relationship between the 120 NFMGs and NF-*κ*B transcription factor. (d) The expression of the 120 NFMGs in each TCGA sample was shown in heatmap. (e, f) GO analysis of (e) upregulated NFMGs and (f) downregulated NFMGs. (g) KEGG analysis of the 120 NFMGs. NFMGs: NF-*κ*B-targeted metabolic genes; TCGA: The Cancer Genome Atlas; DEGs: differential expressed genes; MSigDB: the Molecular Signatures Database; GO: Gene Ontology; KEGG: Kyoto Encyclopedia of Genes and Genomes.

DEGs (*n* = 4975) with protein coding function have been identified between normal and tumor samples from the GC RNA-seq of TCGA, which included 3908 upregulated DEGs and 1067 downregulated DEGs (Figure 2(a)). To identify NFMGs, we dug three gene cohorts: first cohort consisted of 2927 NF-*κ*B-related DGEs that were confirmed by Pearson correlation analysis; second cohort comprised of 26671 NF-*κ*B-targeted genes that were downloaded from the hTFtarget database; third cohort included 948 metabolic genes from the MSigDB database (Figure 2(b)). The overlapped part of these three cohorts was considered as the NFMGs, containing 110 upregulated and 10 downregulated genes. The Sankey diagram displayed the targeted interaction between NFMGs and NF-*κ*B transcription factors, and the expression of NFMGs was also showed by heatmap (Figures 2(c) and 2(d)). GO analysis indicated that the upregulated NFMGs were mainly enriched in amino acid or ribonucleotide metabolism-related biological process, while downregulated NFMGs focused on lipid metabolism-related biological process (Figures 2(e) and 2(f)). KEGG analysis demonstrated that in the upregulated NFMGs, the majority enriched pathways were also concerned in amino acid or ribonucleotide-related metabolism pathway, including the metabolism of purine and pyrimidine, as well as several essential amino acids (Figure 2(g)). And the downregulated genes were mainly enriched in arachidonic acid metabolism, pyruvate metabolism, and regulation of lipolysis in adipocytes (Figure 2(g)).

### 3.3. Consensus Clustering Based on NFMGs

**Figure 3:**
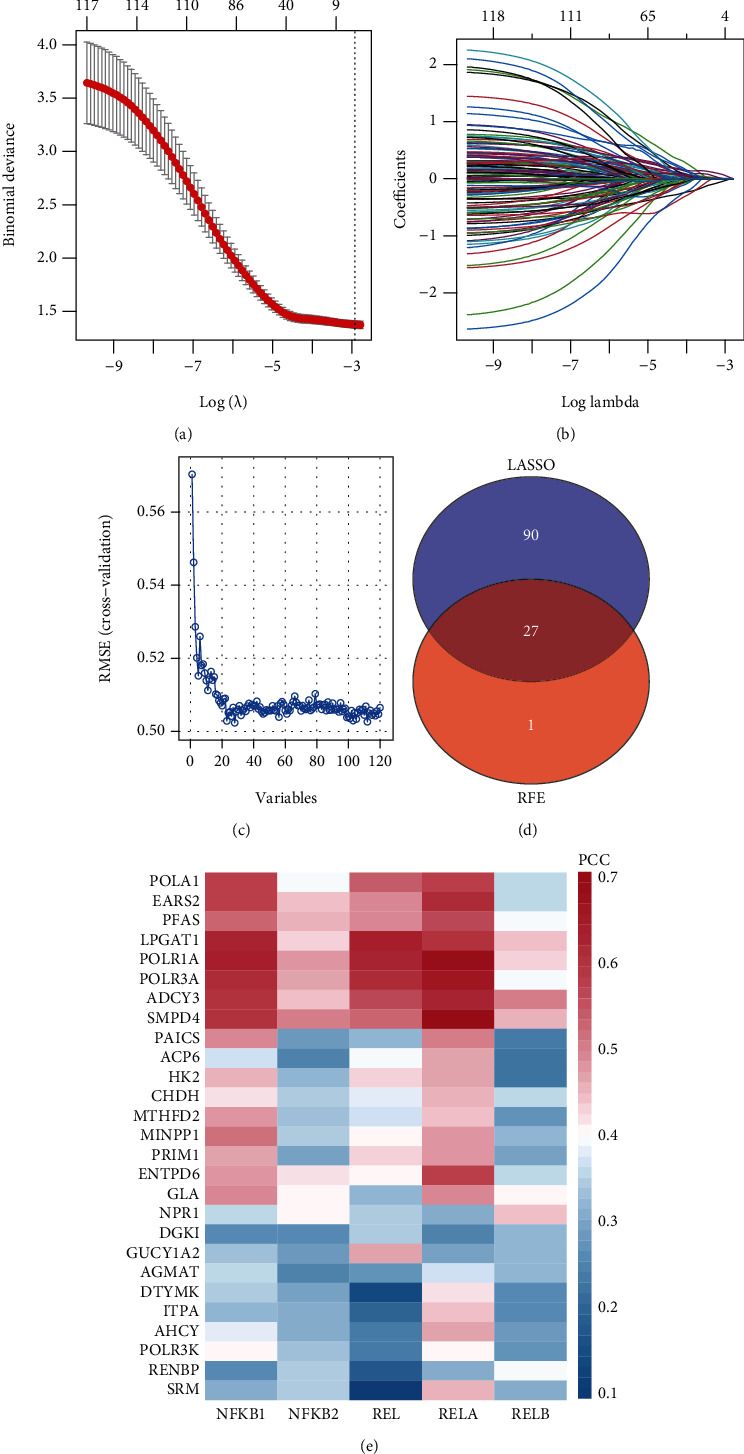
Identification of candidate NFMGs. (a) Cross-validation for tuning parameter screening in the LASSO regression model. (b) Log (lambda) value of the 27 NFMGs in the LASSO regression model. (c) Parameter diagram of the RFE algorithm. The smaller the RMSE, the less the error of the RFE model. The corresponding genes with least RMSE were selected. (d) Venn diagram showed the overlapped NFMGs between LASSO and RFE algorithms. (e) Pearson correlation analysis between the candidate NFMGs and NF-*κ*B transcription factor. NFMGs: NF-*κ*B targeted metabolic genes; LASSO: least absolute shrinkage and selection operator; RFE: recursive feature elimination; RMSE: root mean square error; adj. *p*. val: adjusted *p* value; PCC: Pearson correlation coefficient.

**Figure 4:**
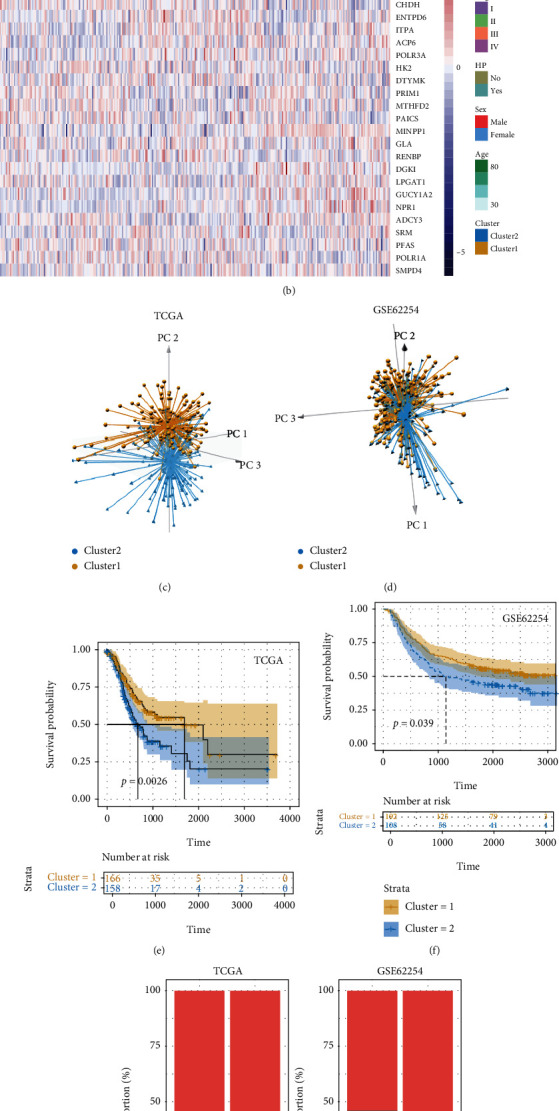
Unsupervised clustering of GC based on the 27 NFMGs. (a, b) Consensus clustering sorts GC samples into two clusters in (a) TCGA and (b) GSE62254 datasets. (c, d) Principal component analysis of the two clusters in (c) TCGA and (d) GSE62254 datasets. (e, f) Survival analysis of the two clusters in (e) TCGA and (f) GSE62254 datasets. (g–i) Proportion of the clinical features in the two clusters in TCGA and GSE62254 datasets. GC: gastric cancer; NFMGs: NF-*κ*B-targeted metabolic genes; TCGA: The Cancer Genome Atlas.

According to the survival data, the number of NFMGs was reduced by using LASSO and RFE algorithms. The parameters of LASSO and RFE were also displayed (Figures 3(a)–3(c)). Finally, 117 and 28 genes were identified by LASSO and RFE algorithms, respectively, and 27 overlapped genes were served as the candidate NFMGs (Figure 3(d)). The correlation of these NFMGs and each NF-*κ*B transcription factor was shown in the heatmap (Figure 3(e)). Subsequently, consensus clustering was performed in TCGA and GSE62254 chip independently (Figures 4(a) and 4(b)). Considering the cluster based on NFMGs, GSVA was employed to analyze the enriched metabolic pathways in the two clusters. Our data showed that cluster 1 had more enriched metabolic pathways than cluster 2. Particularly, amino acid, lipid, and nucleotide as well as glucose-related metabolic pathways were significantly enriched in cluster 1, while few metabolic pathways, such as glycosphingolipid-related pathways, were enriched in cluster 2 (Figure S2). Principal component analysis (PCA) indicated that the two clusters were well classified (Figures 4(c) and 4(d)). Survival analysis demonstrated that patients in cluster 1 showed more favourable survival than patients in cluster 2 (Figures 4(e) and 4(f)). In addition, the proportion of cluster 1 and cluster 2 in different age, sex, or tumor stage has no significant difference (Figures 4(g)–4(i)).

### 3.4. Tumor Microenvironment and Immune Infiltration Assessment across Two Clusters

**Figure 5:**
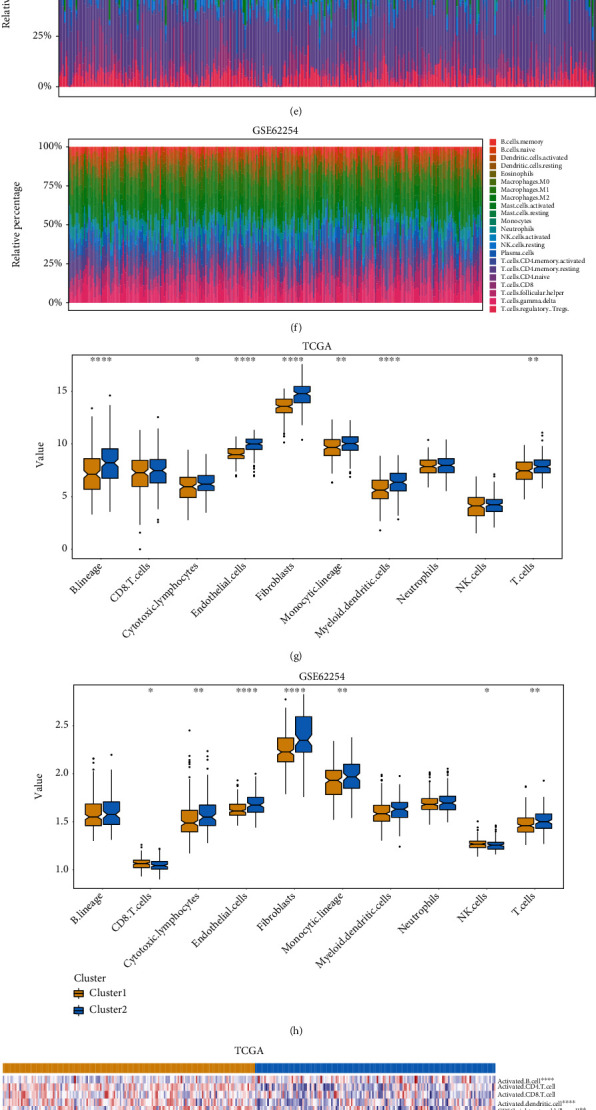
Tumor microenvironment and immune infiltration assessment in the two clusters. (a–d) Estimate analysis in TCGA and GSE62254 datasets. (e, f) The proportion of 22 immune cells in the two clusters was calculated by the CIBERSORT algorithm in (e) TCGA and (f) GSE62254 datasets. (g, h) The MCPcounter algorithm showed that there is more immune infiltration in cluster 2. (i, j) ssGSEA revealed the immune infiltration in the two clusters in (i) TCGA and (j) GSE62254 datasets. ^∗^*p* < 0.05, ^∗∗^*p* < 0.01, ^∗∗∗^*p* < 0.001, and ^∗∗∗∗^*p* < 0.0001. TCGA: The Cancer Genome Atlas; ssGSEA: Single-sample Gene Set Enrichment Analysis.

The ESTIMATE algorithm was further used to evaluate the tumor microenvironment between two clusters. As shown, cluster 2 had higher stromal (*p* < 0.0001), immune (*p* < 0.01), and ESTIMATE (*p* < 0.0001) scores than cluster 1 in both TCGA and GSE62254 datasets, while cluster 2 had lower tumor purity than cluster 1 (*p* < 0.0001) (Figures 5(a)–5(d)). Lower tumor purity usually implied higher immune infiltration. We next applied CIBERSORT, MCPcounter, and ssGSEA algorithms to determine the abundance of different immune cells. Comprehensive consideration of these analysis indicated that cluster 2 had a higher immune infiltration than cluster 1 (Figures 5(e) and 5(f)). The MCPcounter algorithm showed that the abundance of T cell, cytotoxic lymphocyte, monocytic lineage, endothelial cell, and fibroblast was significantly elevated in cluster 2 (Figures 5(e) and 5(f)). The ssGSEA algorithm revealed that majority immune cells, such as effector memory CD4 T cell, effector memory CD8 T cell, natural killer cell, plasmacytoid dendritic cell, and Tregs, were significantly enriched in cluster 2, while memory B cell was enriched in cluster 1 (Figures 5(e) and 5(f)). In addition, we calculated the TMB value of each sample and found that cluster 1 possessed higher TMB than cluster 2 (Figure S3(a)). Intriguingly, we also observed 47.6% samples in cluster 1 had high/low microsatellite instability (MSI-H/L), while only 15.9% samples in cluster 2 had (*p* < 0.0001) (Figure S3(b)). These data suggested that cluster based on NFMGs could achieve a distinct subtype of GC, associated with prognosis, tumor microenvironment, and immune infiltration.

### 3.5. The Expression of Immune Checkpoint Genes (ICGs) and Immunotherapy Sensitivity

**Figure 6:**
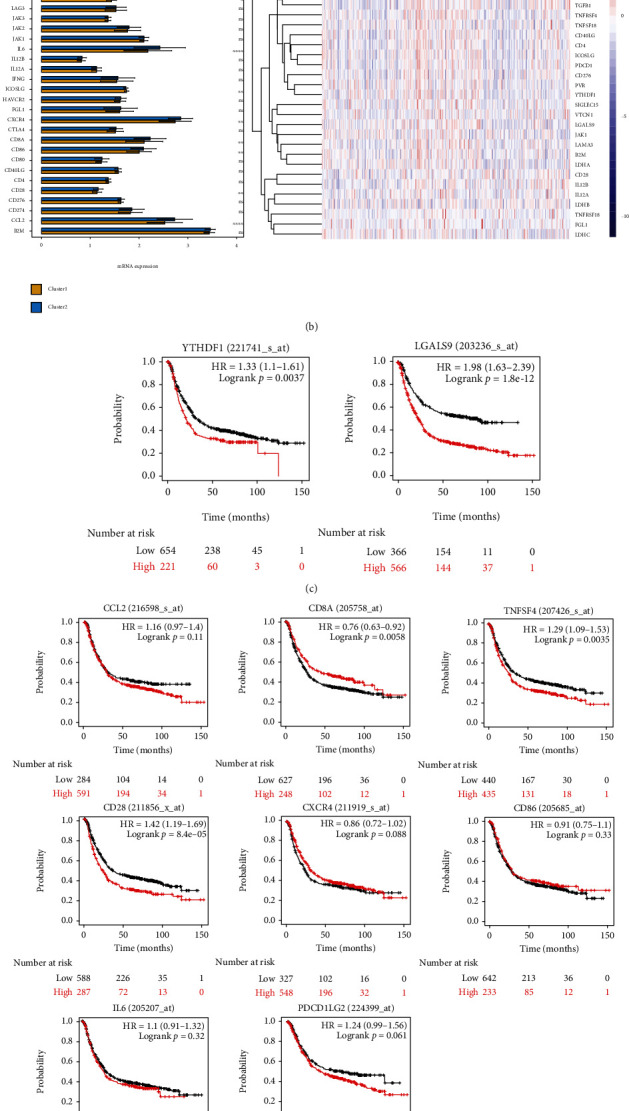
The expression of ICGs in the two clusters. (a, b) Differential analysis of the 42 ICGs in the two clusters. The expression of these ICGs was shown as heatmap. (c) Kaplan-Meier plot of *YTHDF1* and *LGALS9* in GC. (d) Kaplan-Meier plot of *CCL2*, *CD8A*, *CD28*, *CXCR4*, *IL6*, *PDCD1LG2*, *PTPRC*, *TGFB1*, *TNFSF4*, and *CD86* in GC. (e) Kaplan-Meier plot of *TNFSF18* and *TNFRSF18* in GC. ^∗^*p* < 0.05, ^∗∗^*p* < 0.01, ^∗∗∗^*p* < 0.001, and ^∗∗∗∗^*p* < 0.0001. ICGs: immune checkpoint genes; GC: gastric cancer.

**Figure 7:**
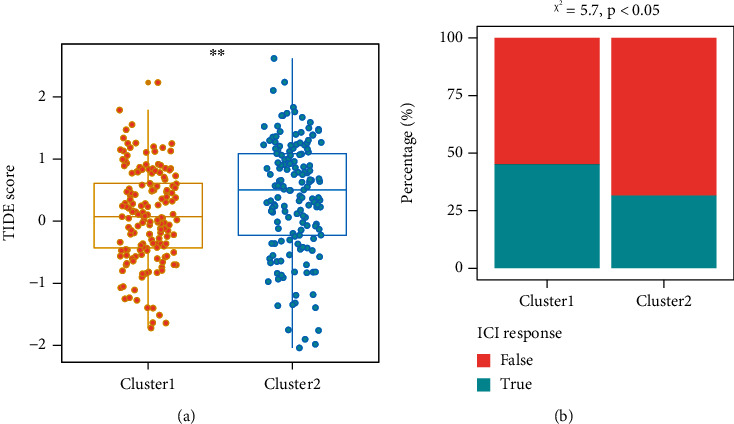
TIDE score predicted the ICI sensitivity in the two clusters. (a) The TIDE score between cluster 1 and cluster 2. (b) The TIDE algorithm showed differential ICI sensitivity in the two clusters. ^∗∗^*p* < 0.01. TIDE: tumor immune dysfunction and exclusion; ICI, immune checkpoint inhibitor.

According to previous study, a total of 42 ICGs were selected for further analysis [37–45]. Overall, we observed more overexpressed ICGs in cluster 2 than that in cluster 1, while the distribution of ICGs in TCGA and GSE62254 datasets was slightly different (Figures 6(a) and 6(b)). Based on the two datasets, only 2 ICGs (*YTHDF1* and *LGALS9*) were significantly overexpressed in cluster 1, and upregulation of them could predict poor prognosis (Figure 6(c)). More ICGs (*CCL2*, *CD8A*, *CD28*, *CXCR4*, *IL6*, *PDCD1LG2*, *PTPRC*, *TGFB1*, *TNFSF4*, and *CD86*) were significantly overexpressed in cluster 2, among which *CD28*, *PTPRC*, *TGFB1*, and *TNFSF4* were associated with poor prognosis (Figure 6(d)). Of note, the expressions of *TNFSF18* and *TNFRSF18* were various in two datasets, while they were significantly associated with GC prognosis (Figure 6(e)). TIDE score was next used to predict the response to ICI. The number of patients that displayed positive response to ICI in cluster 1 was higher than that in cluster 2 (45.2% vs. 31.6%, *p* < 0.05) (Figure 7 and Figure S4). These data demonstrated that cluster 2 had higher ICGs but a less positive response to ICI, which may be responsible for the poor prognosis.

### 3.6. Generation and Validation of Risk Score and Nomogram

**Figure 8:**
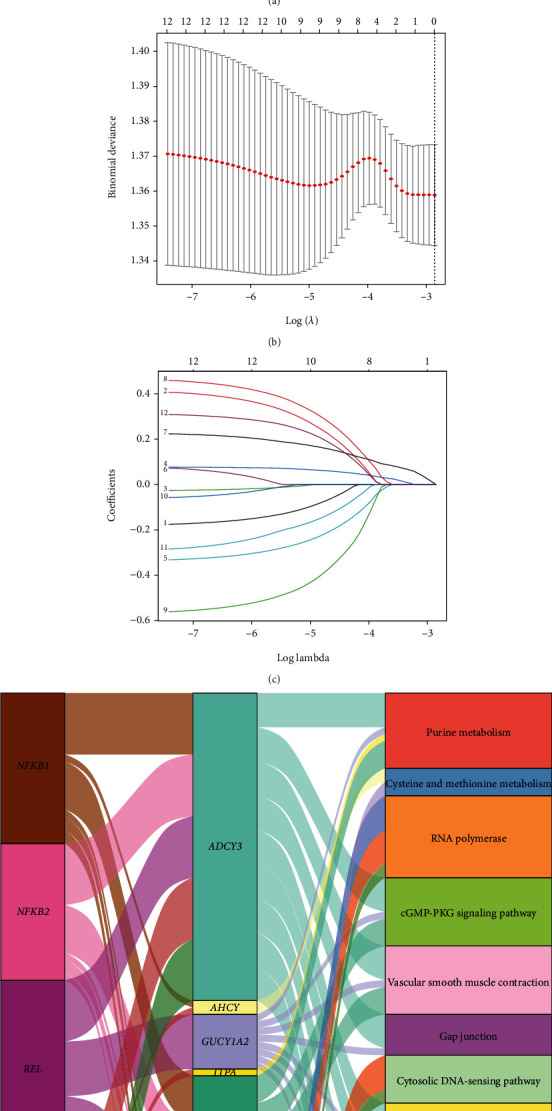
Screening of NFMGs with prognostic signature. (a) The PPI network and MCODE analysis. (b, c) Parameters of the LASSO regression model. (d) The Sankey plot displayed the targeted relationship between NF-*κ*B transcription factors and prognostic NFMGs, as well as KEGG analysis of these NFMGs. NFMGs: NF-*κ*B-targeted metabolic genes; PPI: protein-protein interaction; LASSO: least absolute shrinkage and selection operator; KEGG: Kyoto Encyclopedia of Genes and Genomes.

**Figure 9:**
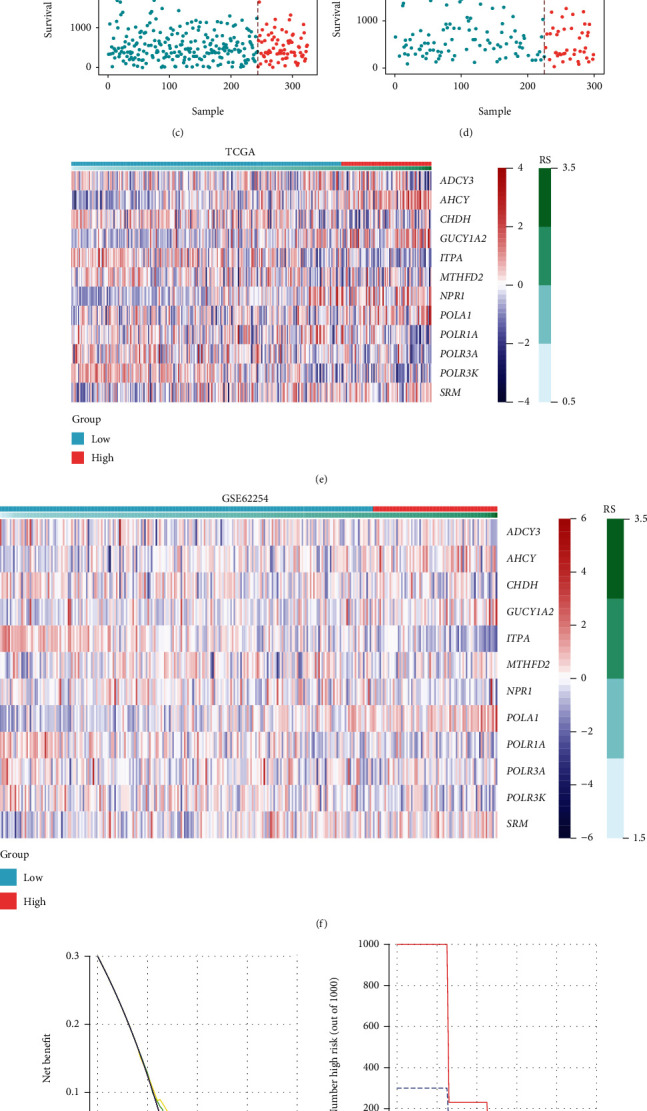
Performance assessment of the RS for survival predication in training and validation set. (a, b) Survival analysis of the RS in (a) TCGA and (b) GSE62254 datasets showed that the low-risk group had a longer OS than the high-risk group. (c, d) RS was calculated in (c) TCGA and (d) GSE62254 datasets and displayed as scatter diagram. (e, f) The expression of the related NFMGs in (e) TCGA and (f) GSE62254 datasets. (g, h) DCA analysis was used to evaluate the efficacy of the RS in (g) TCGA and (h) GSE62254 datasets. RS: risk score; TCGA: The Cancer Genome Atlas; OS: overall survival; NFMGs: NF-*κ*B-targeted metabolic genes; DCA: decision curve analysis.

**Figure 10:**
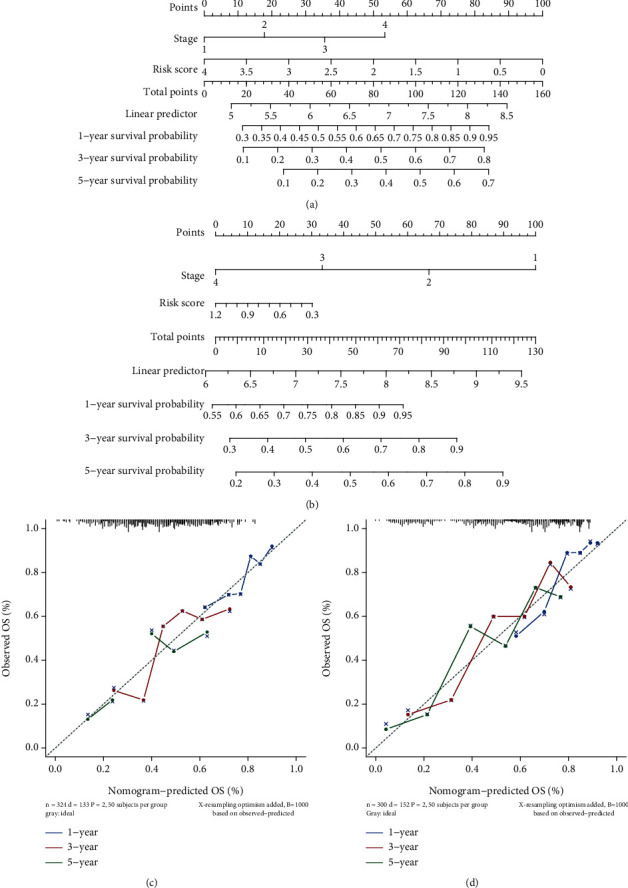
Construction of nomogram. (a, b) Nomogram was used to predict 1-year, 3-year, and 5-year OS in (a) TCGA and (b) GSE62254. (c, d) The calibration curves for predicting 1-year, 3-year, and 5-year OS in (c) TCGA and (d) GSE62254. OS: overall survival.

The protein-protein interaction (PPI) network of the 27 candidate NFMGs was analyzed by using the STRING online tool and visualized by the Cytoscape software (Figure 8(a)). Two modules were further identified by using MCODE app, among which 12 NFMGs were selected for next analysis (Figure 8(a)). To construct a RS for predicting survival, the LASSO algorithm was employed again and calculated a formula as follows: RS = (−0.1751 × Exp *ADCY*3) + (0.4069 × Exp *AHCY*) + (−0.0259 × Exp *CHDH*) + (0.0766 × Exp *GUCY*1*A*2) + (−0.3318 × Exp *ITPA*) + (0.0723 × Exp *MTHFD*2) + (0.2233 × Exp *NRP*1) + (0.4586 × Exp *POLA*1) + (−0.5607 × Exp *POLR*1*A*) + (−0.0574 × Exp *POLR*3*A*) + (−0.284 × Exp *POLR*3*K*) + (0.3087 × Exp *SRM*). The parameters of the LASSO algorithm were also shown (Figures 8(b) and 8(c)). Function enrichment analysis of these 12 genes suggested that they were related to purine metabolism, cysteine, and methionine metabolism (Figure 8(d)). The RS of each sample was further calculated. Setting interquartile as the cutoff value, patients were divided into low-risk and high-risk groups. Survival analysis suggested that patients in the high-risk group may suffer a shorter OS than patients in the low-risk group in training cohort (TCGA dataset) and validation cohort (GSE62254 dataset) (Figures 9(a) and 9(b)). The OS of patients was gradually decreased along with the increase of RS, and the expression of each related NFMG was shown in the heatmap (Figures 9(c)–9(f)). We next applied DCA curve aiming to compare the clinical efficacy among RS and clinical characteristics. In training cohort, RS possessed the most effective prediction capability, and in validation cohort, the prediction efficacy of RS was second only to stage (Figures 9(g) and 9(h)). To further improve the clinical application, a nomogram was according to RS and stage in both training and validation cohorts (Figures 10(a) and 10(b)). The calibration plots indicated that the nomogram had a well predictive efficacy for GC patients' 1-year, 3-year, and 5-year OS rates when compared with an ideal model in training and validation cohorts (Figures 10(c) and 10(d)).

## 4. Discussion

Deregulating cellular metabolism and avoiding immune destruction are important hallmarks for tumorigenesis and development [9]. In this paper, we identified a set of metabolic genes that may be regulated by NF-*κ*B transcription factors. Based on these NFMGs, we applied an unsupervised clustering method to uncover a novel subtype of GC. Among the subtypes, cluster 2 had poor prognosis, low tumor purity, and enriched immune characteristics. Of note, cluster 2 possessed lower TMB, MSI, and response rate to ICI than that in cluster 1. Finally, we constructed a risk score according to NFMGs, which possessed an outstanding efficacy for predicting OS in GC patients.

Since discovery, NF-*κ*B family was always the research focus as classical transcription factors. Early study revealed that NF-*κ*B proteins can accelerate cell proliferation, inhibit apoptosis, promote cell migration and invasion, and stimulate angiogenesis in tumorigenesis and development [8]. In 2004, two seminal studies uncovered that NF-*κ*B proteins acted as a molecular lynchpin linking inflammation to cancer in inflammation­driven colorectal cancer and hepatocellular carcinoma [46, 47]. Subsequently, development in cancer genetics and genomics as well as identification of a novel generation of cancer hallmarks conferred the discovery of novel NF-*κ*B-dependent cancer vulnerabilities [6]. Among those, the centre of attraction is the intricate entwine between NF-*κ*B and reprogramming of energy metabolism or evasion from immune surveillance [6]. As referred, aberrant metabolism has intricate influence on cancer immunity: for one thing, high metabolic activity of cancer cells conduces to a nutrient deficient and hypoxic microenvironment, leading to metabolic competition with infiltrating immune cells; for another, aberrant metabolism in immune cells also regulates immune cell function [10]. Here, we identified 120 NFMGs in GC. These genes may be the target genes of NF-*κ*B transcription factors and could regulate metabolic progress. Functional enrichment analysis indicated that these NFMGs were mainly enriched in amino acid, lipid, and nucleotide-related metabolic pathways, which were closely associated with cancer immune cells [10]. Consequently, how NF-*κ*B transcription factors mediated cancer immunity via regulating metabolism may be revealed by the further study of these NFMGs.

Cancer classification based on clinical characteristics or gene expression features is of great importance in predicting prognosis and guiding therapy. More and more precise and diversified oncotherapy weakens the advantage of traditional cancer classification. Of note, increasing gene sets with specific function were employed for typing cancer. In hepatocellular carcinoma, 41 ferroptosis-related genes were adopted to divide patients into two phenotypes: Ferroptosis-L phenotype and Ferroptosis-H phenotype, in which Ferroptosis-H phenotype had a worse OS than that in Ferroptosis-L phenotype (median OS: 3.11 vs. 6.93 years, *p* < 0.001) [48]. Glioma was classified into low and high hypoxia risk groups according to hypoxia signature, which was also associated with patients' OS [49]. Moreover, previous research used various gene sets, including glycolysis-related gene set, cell cycle-related gene set, angiogenesis-related gene set, and N6-methyladenosine methylation gene set, to achieve classification of GC [50–53]. All of these classification could effectively forecast patients' prognosis. Therefore, the transcriptome data of GC cases were investigated aiming to identify NFMG-related classification. Indeed, this GC classification was significantly associated with patients' OS, in which cluster 1 possessed better prognosis.

Further analysis revealed that cluster 1 was a characteristic of high active metabolism and low immune infiltration, consistent with previous study [10]. In turn, cluster 2 was characterized by high immune infiltration. Unfortunately, however, these infiltrating immune cells did not bring a better outcome for cluster 2, which could be explained by the following reasons. Firstly, except antitumor immune cells (CD8 T cell, memory CD8 T cell, and NK cell), several immunosuppressive cells, such as Tregs and myeloid-derived suppressor cells (MDSCs), were also enriched in cluster 2. Secondly, more reported immune checkpoints were overexpressed in cluster 2, which may depress the activity of infiltrating immune cells. Thirdly, cluster 1 was involved in more amino acid metabolism, including serine, cysteine, leucine, and arginine. Leucine was necessary for effector function and proper differentiation in effector CD8^+^ and CD4^+^ T cells [54]; arginine was beneficial for T cell survival and antitumor functionality [55]; cysteine was required for T cells during antigen presentation and subsequent T cell activation [56]; serine as a key immunometabolite could regulate T cell proliferative capacity [57]. These data hinted that low infiltration immune cells in cluster 1 may have certain antitumor activity due to the active metabolism.

As abovementioned, guiding treatment accounts for the major purpose of cancer classification. TIDE score was an excellent method for predicting response to ICI, especially to anti-PD1 or anti-CTLA4 therapy—the main ICI therapy in clinic [29, 58]. We found that cluster 1 had higher response rate to ICI than cluster 2. From the ICG expression profile, PD1, PD-L1, and CTLA4 had no significant difference between the two clusters, while several other immune checkpoints were enriched in cluster 2. Additional inhibitory checkpoints were considered as the reason of cancer's resistance to ICI [59]. In addition, we observed higher TMB and higher proportion of MSI-H/L in cluster 1 than in cluster 2. Previous study indicated that MSI cancers have a higher antitumor activity of ICI therapy, and TMB is also associated with improved survival in patients receiving ICI across a wide variety of cancer types [60, 61]. Therefore, these additional inhibitory checkpoints, low TMB, and MSI may contribute to the low response rate of cluster 2 to ICI in GC.

Previous paper also studied the metabolism-related genes (MRGs) in GC and identified a 13-MRG risk model with prognostic signature [62]. The different is that we only focused on NF-*κ*B transcription factors targeted metabolic genes. Here, we constructed a 12-NFMG RS with prognostic signature based on the LASSO regression analyses, in which the high-risk group had a significantly shorter OS than the low-risk group. We also established a nomogram according to the RS and GC stage and followed the nomogram was verified to have a well performance in predicting patients' OS. Reviewing the 12 NFMGs, *GUCY1A2* has been reported to be an independent prognostic marker for GC [63]; single-cell RNA sequencing on gastric hepatoid adenocarcinoma indicated that *AHCY* may be potential targets for its treatment [64]; *MTHFD2*-encoded key enzyme in folate metabolism and methyl donor SAM production and its knockdown significantly suppressed GC cell proliferation [65]; *ADCY3*-encoded protein may exert its tumor-promoting effects via the cAMP/PKA/CREB pathway [66]; *POLR1A* has also been identified to be associated with prognosis in GC [62]; *NRP1*-encoded protein could accelerate cell proliferation, invasion, and migration in GC [67, 68]. The rest involved genes, including *POLR3K*, *POLR3A*, *ITPA*, *POLA1*, *CHDH*, and *SRM*, have not been reported in GC, providing multiple research objectives for exploring the underlying mechanisms of NF-*κ*B-mediated metabolism.

Several limitations should also be mentioned. First of all, the same metabolism in cancer cells or immune cells makes different influence on tumor immune microenvironment [10]. However, our data were from the simple RNA-seq data of tumor tissues, and whereby it is hard to clarify the intricate tumor microenvironment. Further application of single-cell sequencing technique or spatial transcriptome may better uncover the characteristics and regulatory mechanisms of GC's microenvironment. What is more, the targeted regulation of NF-*κ*B transcription factors against these NFMGs was only predicted by using online dataset, which should be further confirmed by using protein-nucleic acid interaction assays, such as luciferase reporter gene assay, chromatin immunoprecipitation, and electrophoretic mobility shift assays. Last but not the least, the clinical application of this classification and RS for GC should also be further testified by more clinical data.

## 5. Conclusion

In summary, we used NFMGs to cluster GC samples into two subtypes that possessed significantly different metabolism, immune infiltration, TMB, and microsatellite status. These subtypes also had different response to ICI therapy, which may provide better individualized regimens for GC's ICI therapy. We further established a risk score based on 12 NFMGs, and this score could effectively predict GC patients' OS. Further expanding of the population data for validation may facilitate the clinical application of our models in GC. In addition, in depth study of these NFMGs would contribute to further understanding of the link between metabolism and immunity in GC, as well as its underlying mechanisms.

## Figures and Tables

**Table 1:** Characteristics of GC patients in TCGA and GSE62254 datasets.

	TCGA (*n* = 324)	GSE62254 (*n* = 300)
Age		
≤65	144	172
>65	180	128
Sex		
Male	206	199
Female	118	101
Grade		
G1	8	
G2	115	
G3	201	
Stage		
I	43	30
II	107	97
III	142	96
IV	32	77
Pathology		
Adenocarcinoma	178	71
Mucinous adenocarcinoma	17	3
Papillary adenocarcinoma	4	8
Tubular adenocarcinoma	61	141
Signet ring cell carcinoma	10	42
Other	54	35
HP infection		
No	131	72
Yes	16	55
Recurrence		
No	198	157
Yes	54	125

## Data Availability

The RNA-seq data and related clinical data were presented in TCGA (https://gdc-portal.nci.nih.gov/) and GSE62254 (https://www.ncbi.nlm.nih.gov/geo/query/acc.cgi?acc=GSE62254). The expression of NF-*κ*B transcription factors was downloaded from TIMER 2.0 (http://timer.comp-genomics.org/) and HPA (https://www.proteinatlas.org/) datasets. We sourced the survival analysis of NF-*κ*B transcription factors from the Kaplan-Meier Plotter online tool (http://kmplot.com/analysis/index.php?p=background). The response rate to ICI was predicted by using the TIDE dataset (http://tide.dfci.harvard.edu/).

## Supplementary Materials

Supplementary 1 Supplemental file 1: R codes.

Supplementary 2 Supplemental file 2: supplemental figures and related legends.
