# Supplementary material for: NF-κB-Related Metabolic Gene Signature Predicts the Prognosis and Immunotherapy Response in Gastric Cancer
Source: Biomed Res Int. 2022 Jan 4;2022:5092505. doi: 10.1155/2022/5092505 (PMC8753254; doi:10.1155/2022/5092505)
Supplement: Supplementary 2 — Supplemental file 2: supplemental figures and related legends. [file 5092505.f2.docx]

**Supplemental Figure Legends**

**Figure S1. The expression of NF-κB transcriptional factors in GC tissues.** Immunohistochemistry data obtained from HPA database showed the positive expression of NF-κB1, NF-κB2, RelA, RelB as well as c-Rel in GC tissues. GC, gastric cancer; HPA, the Human Protein Atlas.

**Figure S2. Metabolic pathway analysis in the two clusters.**

**Figure S3. TMB and microsatellite status evaluation in the two clusters.** (A) TMB assessment showed that cluster2 had lower TMB than cluster1. (B) More samples in cluster1 occurred MSI-H/L than that in cluster2. **** indicated *p* < 0.0001. TMB, tumor mutant burden; MSI-H/L, high/low microsatellite instability; MSS, microsatellite, stability.

**Figure S4. The TIDE score of each sample in TCGA dataset.**

**Figure S1**

**
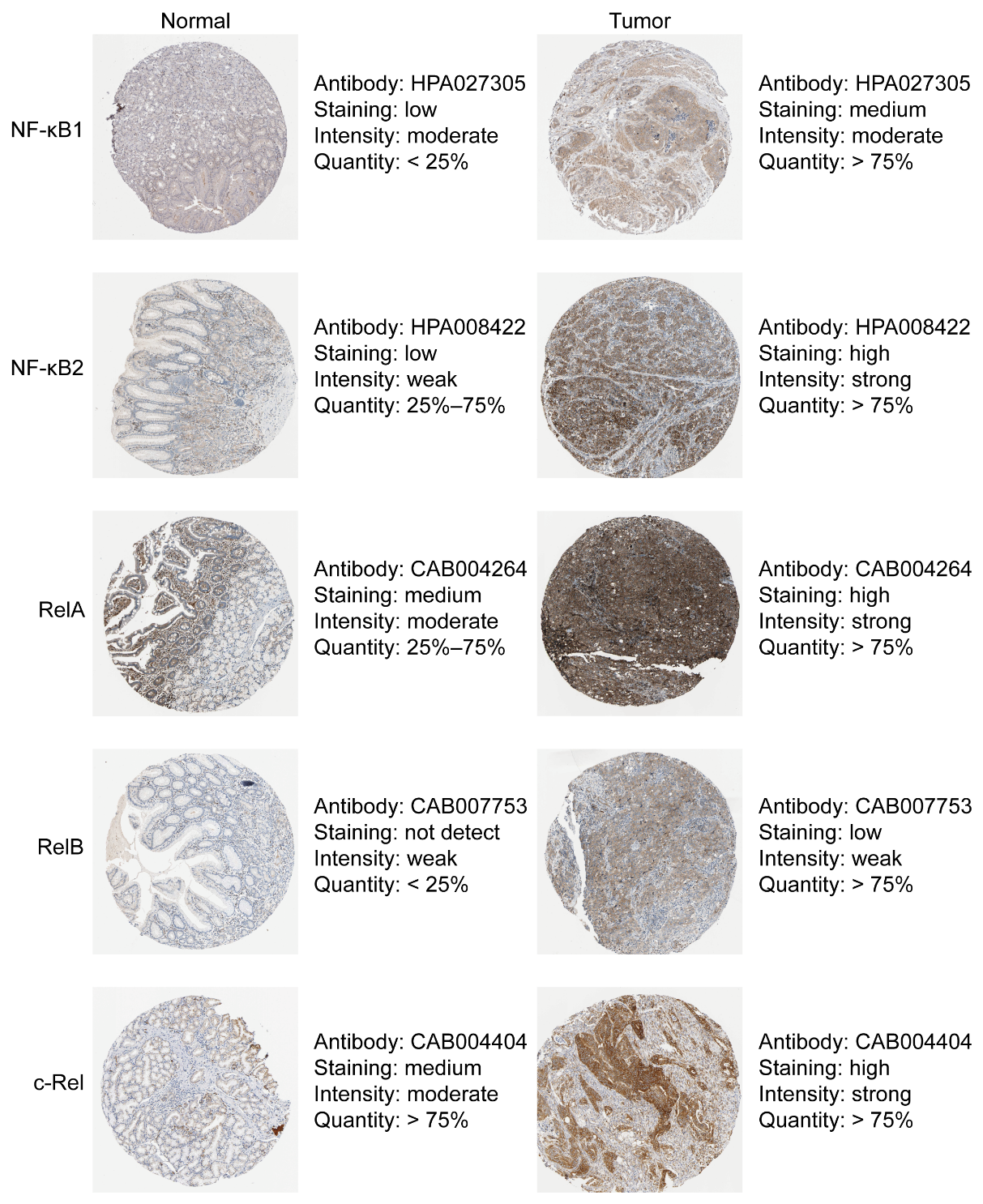
**

**Figure S2**

**
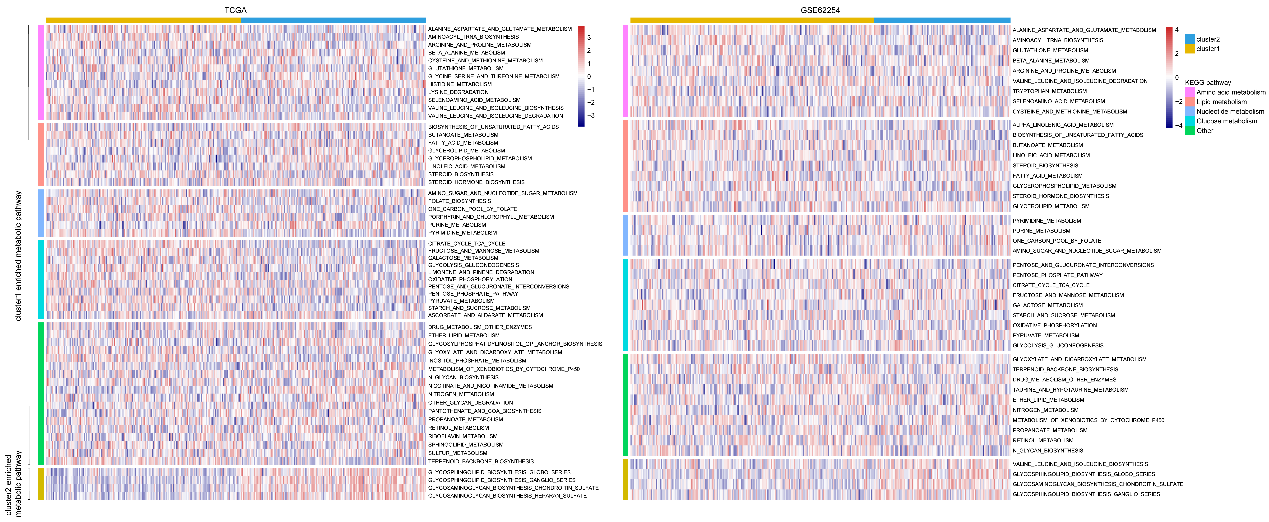
**

**Figure S3**

**
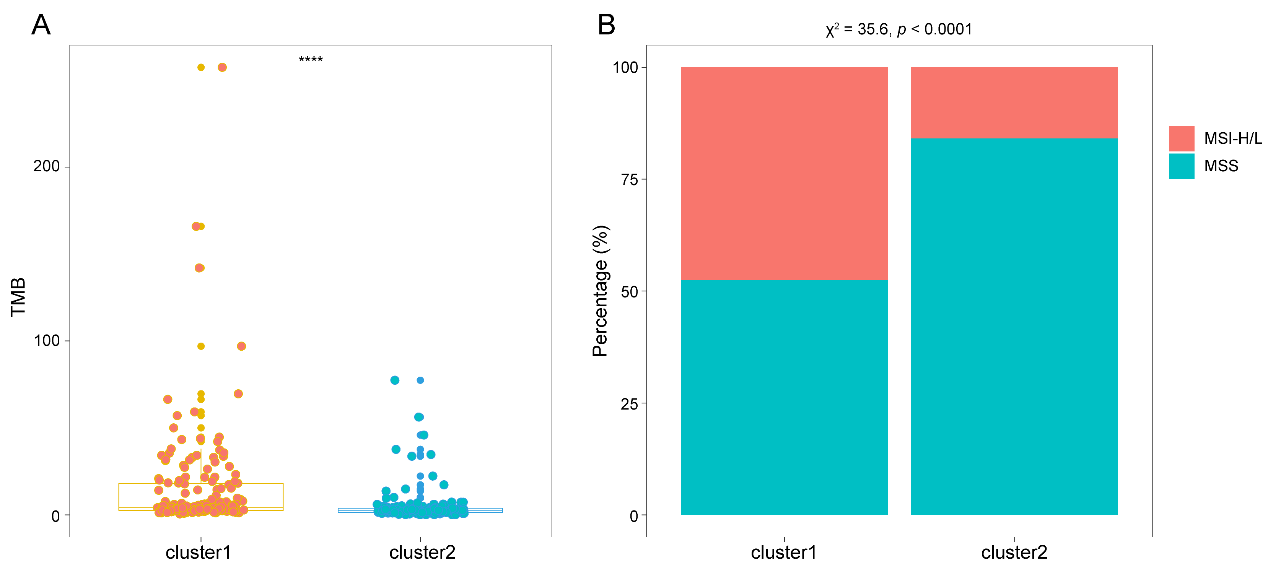
**

**Figure S4**

**
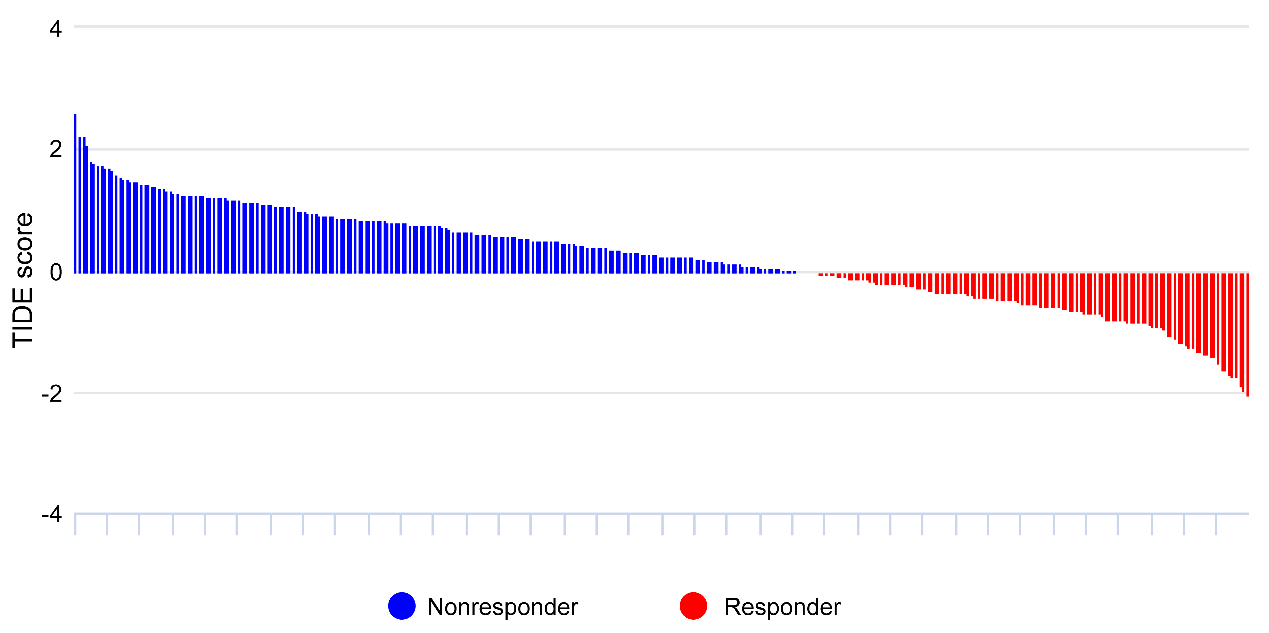
**
